# Trastuzumab-Peptide Interactions: Mechanism and Application in Structure-Based Ligand Design

**DOI:** 10.3390/ijms140816836

**Published:** 2013-08-15

**Authors:** Tian-Yang Sun, Qi Wang, Jin Zhang, Tao Wu, Fan Zhang

**Affiliations:** Soft Matter Research Center, Department of Chemistry, Zhejiang University, Hangzhou 310027, China; E-Mails: suntyy@gmail.com (T.-Y.S.); jin.jean.zhang@gmail.com (J.Z.); fanzhang@cad.zju.edu.cn (F.Z.)

**Keywords:** protein-ligand interaction, binding pocket, binding mechanism, peptide design, molecular dynamics, MM-GBSA

## Abstract

Understanding of protein-ligand interactions and its influences on protein stability is necessary in the research on all biological processes and correlative applications, for instance, the appropriate affinity ligand design for the purification of bio-drugs. In this study, computational methods were applied to identify binding site interaction details between trastuzumab and its natural receptor. Trastuzumab is an approved antibody used in the treatment of human breast cancer for patients whose tumors overexpress the HER2 (human epidermal growth factor receptor 2) protein. However, rational design of affinity ligands to keep the stability of protein during the binding process is still a challenge. Herein, molecular simulations and quantum mechanics were used on protein-ligand interaction analysis and protein ligand design. We analyzed the structure of the HER2-trastuzumab complex by molecular dynamics (MD) simulations. The interaction energies of the mutated peptides indicate that trastuzumab binds to ligand through electrostatic and hydrophobic interactions. Quantitative investigation of interactions shows that electrostatic interactions play the most important role in the binding of the peptide ligand. Prime/MM-GBSA calculations were carried out to predict the binding affinity of the designed peptide ligands. A high binding affinity and specificity peptide ligand is designed rationally with equivalent interaction energy to the wild-type octadecapeptide. The results offer new insights into affinity ligand design.

## 1. Introduction

Investigation of protein-protein and protein-ligand interactions plays a critical role in the understanding of many biological processes [1]. It is also of great importance to many related applications such as biosensors, drug delivery and protein purification [2–4]. Thus, understanding of these biomolecular interactions is one of the essential goals of the physical chemistry and biophysics communities. In the past decades, an increasing number of research articles have been published, enhancing our understanding of protein-protein and protein-ligand interactions [5–7].

Recent technological advances have confirmed that biomolecules such as monoclonal antibodies could play important roles not only as therapeutic agents for a wide variety of diseases, but also as agents used in imaging or diagnostic applications. For example, the humanized anti-HER2 (human epidermal growth factor receptor 2) receptor monoclonal antibody, trastuzumab (Herceptin^®^), was the first antibody approved by the U. S. Food and Drug Administration for the treatment of metastatic breast cancer. [8,9], and also has a significant therapeutic effect on patients with advanced gastric cancer [10] resulting in HER2 overexpression. Affinity chromatography [11] is generally used for the isolation and purification of proteins and antibodies. Biospecific ligands that are mainly used now (Protein A or G immobilized on appropriate supports as selective ligands) suffer from several drawbacks, such as the complexity of isolation and purification from microbial extracts, the presence of biological contaminants that are difficult to eliminate, the tendency to lose selectivity because of harsh elution (pH 3), washing and sterilization conditions, the high cost and the low stability of sanitation agents used for pyrogen removal [12]. As a result, there is a great deal of interest in the use of small peptide ligands for antibody purification due to their advantages of lower cost, better chemical and biological stability, milder reaction conditions than with large protein ligands and reduced immunogenicity. The development of combinatorial peptide libraries such as phagedisplay technology [13–15] and one-bead-one-peptide combinatorial library methods [16,17] have greatly contributed to the implementation of high-throughput screening in academic laboratories. Experimenters can rapidly screen ligands with expected property from millions of synthesized compounds [18,19]. However, there are some limitations that have to be considered, such as the size and quality of libraries, the large amount of experiments, further mutagenesis and screening of peptides [20].

Computational techniques are used widely to investigate, analyze and predict protein-ligand interactions. They are also used to design new ligands for a variety of proteins in order to reduce expensive experimental efforts [21–23]. However, it is still a challenge to design a ligand efficiently. Several designed ligands, which were expected to be high-affinity to the periplasmic binding protein (PBP), were investigated by Schreier *et al.* [24]. It was found that the investigated proteins did not bind these ligands as expected because of large conformational change of PBP induced by the ligands. Other works also suggest that the protein structure and its flexibility in the binding site can influence the outcome of docking dramatically [25]. These findings indicate that general computational methods need to be revisited and improved for these special goals. As an alternative method, atomistic or atomic molecular simulation presents direct approaches to investigate the atomic details of the interactions. It can provide reliable conformational changes in the binding and characteristics of different amino acid groups, hydrogen bonds, *etc*. Freed *et al*. [26] compared the molecular dynamics (MD) based calculations with the NMR spectroscopy of ubiquitin with ligands, and the results suggested that the MD simulations provided a reasonable description of structural aspects of binding, which is in accordance with NMR. In addition, steered molecular dynamics (SMD) simulation provided important qualitative insights into biological problems. Previous works from our group also confirmed that SMD could be used successfully for the protein-interface free energy calculations of adsorption and desorption mechanism, *etc*. [27,28].

The aim of this study was to explore the binding details through structural and energetic analysis of peptide ligand to trastuzumab, and to investigate a structure-based ligand design strategy with trastuzumab as a model protein. Based on the crystal structure of HER2 complex with its antibody trastuzumab, the protein-ligand (substrate and its mutations) interaction mode and energetic details were investigated by MD simulations. The equilibrium state of designed peptide binding to trastuzumab was obtained by constant velocity pulling (PCV) SMD simulation before relaxation by MD simulation. Finally, MM-GBSA binding free energies were calculated for ligands evaluation.

## 2. Results and Discussion

In this study, molecular dynamics simulations of the trastuzumab antigen-binding fragment (Fab) and an octadecapeptide from its receptor HER2 were run firstly to have a brief understanding of the binding. The three-dimensional structure of the complex is from the protein data bank (PDB: 1N8Z). The octadecapeptide was chosen according to the following rules, which could reduce the system size. As shown in the literature, the trastuzumab-binding site of HER2 consists of three loop regions [residues 557–561 (loop 1), 570–573 (loop 2) and 593–603 (loop 3)]. Interactions formed by the first and third loop are primarily electrostatic, whereas the second loop makes mostly hydrophobic contacts, in which loop 1 and loop 2 are closed to each other [29]. Therefore, a wild-type octadecapeptide formed by residues 557–574 of HER2 (sequence: PEADQCVACAHYKDPPFC, renumbered from 1 to 18) with the trastuzumab Fab was chosen as the initial system for trastuzumab-peptide interactions in this work.

### 2.1. Interactions between Fab and Octadecapeptide Ligand

**Figure 1:**
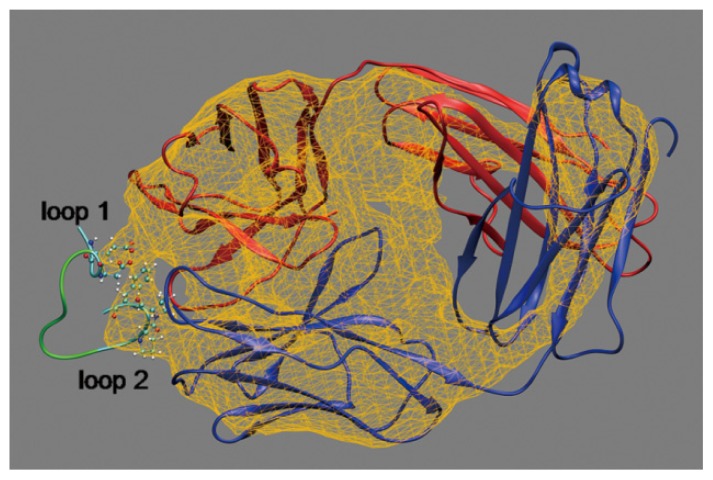
Snapshot of the configuration for the fragment of antigen-binding (Fab) and octadecapeptide system. The red and blue NewCartoon models represent the heavy and light chains of the Fab, respectively. The octadecapeptide in the NewCartoon model contains two loops (cyan) interacting with the Fab and one linking sequence (green). The electrostatic potential isosurface is shown in yellow wireframe. Corey-Pauling-Koltun (CPK) model represents side chains of Glu2, Asp4, Pro15, Pro16 and Phe17 close to the Fab. Water molecules, ions and hydrogen atoms are not displayed for clarity.

The configuration of the Fab-octadecapeptide system surrounded by an electrostatic potential isosurface at −79.86 eV is shown in Figure 1. It shows that the electrostatic potential value outside this surface is lower than the inner part. The external negative electrostatic potential was arisen from the charged residues situated near each other on the interaction interface, indicating that loop 1 and loop 2 are the binding sites, which agrees with the results reported in the literature [29,30]. In order to further explore the molecular nature and mechanism of the interaction between Fab and octadecapeptide ligand, the effect of binding on protein stability, the equilibrated interaction energy, the interaction type and the key residues were investigated.

**Figure 2:**
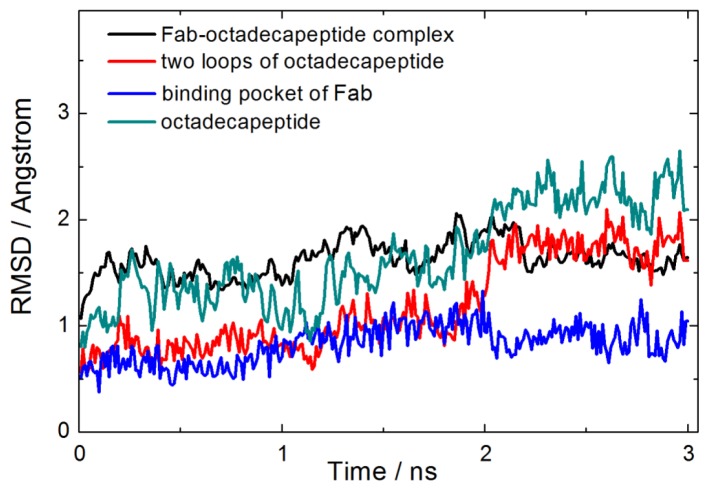
Root mean squared deviation (RMSD) of the backbone of Fab-octadecapeptide complex (black), Fab binding sites (two loop regions) of octadecapeptide (red), octadecapeptide binding groove of Fab (blue), and the octadecapeptide (dark cyan) as a function of simulation time.

Figure 2 displays the root mean squared deviation (RMSD) of the backbone of Fab-octadecapeptide complex, two loop regions of the octadecapeptide, octadecapeptide binding groove of Fab and the octadecapeptide. It is obvious that the conformation of protein-octadecapeptide complex changes slightly, especially after 2.2 ns equilibration. While, the RMSD value of octadecapeptide is the highest one at around 2.2 ± 0.2 Å because of its high flexibility. The RMSD of loop regions is lower than the whole octadecapeptide. Furthermore, the RMSD of the groove of Fab is much lower than other ones during the last 2.2–3.0 ns. This indicates that binding of the protein leads to an increase in the conformation stability within these interaction regions. These predictions agree with the experimental findings that the interaction of proteins with ligands often takes place with an increase in protein stability and structural order [31,32]. That is to say, formation of the Fab-octadecapeptide complex makes the binding site, namely, loop regions of the octadecapeptide and the binding groove of Fab in this system more stable than other parts.

**Figure 3:**
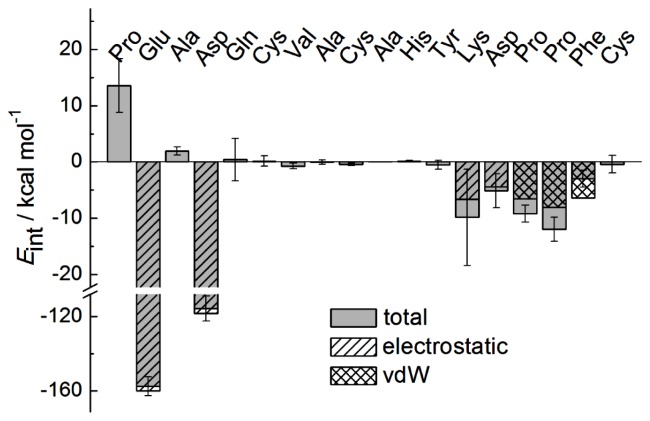
Per-residue interaction energies between octadecapeptide and Fab.

The molecular interactions that contribute to binding and stabilization of the Fab-octadecapeptide complex were obtained on the basis of electrostatic and van der Waals interaction energies per-residue, as shown in Figure 3 (a negative value indicates attraction and a positive value indicates repulsion), which indicates the average interaction energy between each residue of the octadecapeptide and trastuzumab Fab during the last 2.2–3.0 ns equilibrium simulation time. It is obvious that residues with anionic carboxylate side chains such as glutamic acid (Glu2) and aspartic acid (Asp4) from loop 1 performed significant electrostatic interactions of −160.0 kcal·mol^−1^ and −118.3 kcal·mol^−1^, respectively, to which the complex formation could be mainly attributed. This result would be further proved by mutation study in the next section.

**Figure 4:**
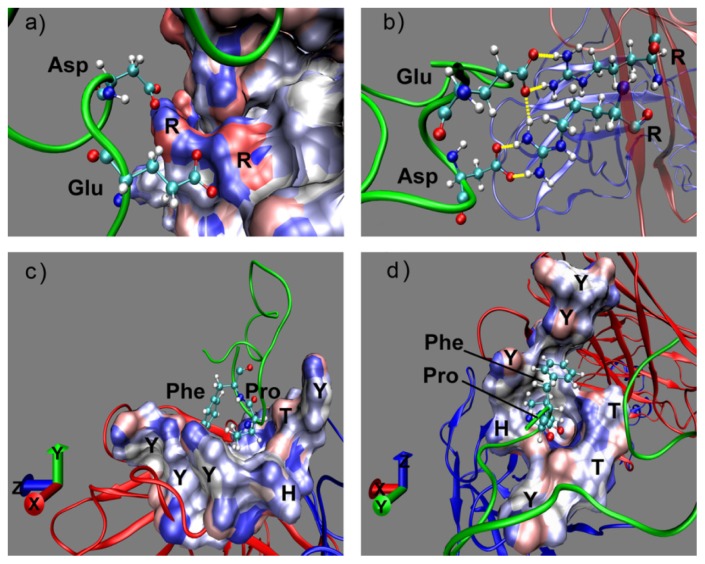
Snapshot of the Fab-octadecapeptide system. (**a**) Electrostatic interaction model; (**b**) Hydrogen bond model between charged residues; (**c**) and (**d**) are side and vertical views of Phe16, Phe17 binding to the Fab hydrophobic groove, respectively. Solvent accessible surface areas colored by electrostatic potential represent Fab binding surfaces. The other part of Fab displayed in the NewCartoon model for clarity. One-letter codes from Fab and three-letter codes from ligand represent particular residues in the interaction.

Moreover, structure analysis suggests that the strong electrostatic interaction between Glu2, Asp4 from the octadecapeptide and Arg50, Arg59 (Figure 4a) with a positively charged guanidino side chain from the Fab heavy chain occur with remarkable hydrogen bond interactions (Figure 4b). The average number of intermolecular hydrogen bonds is 3, when the acceptor was oxygen of carboxylate side chain and the donor was hydrogen of guanidino side chain during the last 2.2–3.0 ns. Here hydrogen bond interaction is a key contributor to the specificity of peptide ligand binding to Fab for its orientation. Figure 4b shows the snapshots of maximum hydrogen bonds occurring between two pairs of charged residue (Glu2, Asp4 and Arg50, Arg59). There are eight terminal hydrogen atoms on these two neighboring guanidyl group of arginine. Therefore, they move and vibrate fast but always within the hydrogen bond distance and the number of hydrogen bond may be formally conserved. In addition to strong electrostatic interaction, obvious van der Waals interactions of hydrophobic residues phenylalanine and proline from loop 2 are also found in Figure 3. Though the interaction energies of Lys13 and Asp14 had almost the same value as Pro15, Pro16 or Phe17, they are attributed to electrostatic interaction at least 10 times smaller than that of Glu2 and even with no selectivity. It shows that Pro16 has the largest van der Waals interaction. By contrast, Phe17 exhibits a large van der Waals attraction but a Coulomb repulsion with Fab. Both of these residues should be considered in the ligand design as a result of these properties. Figure 4c,d shows that Phe17 and Pro16 binding into a groove on the Fab surface were mainly made up of strong hydrophobic and aromatic residues, Tyr and Trp. The hydrophobic binding groove is obtained by searching within 5 angstrom of Pro16 and Phe17 formed by Tyr33, Tyr52, Trp99, Gly103, Tyr105 from Fab heavy chain (chain H) and His91, Tyr92, Thr93, Thr94 from light chain (chain L). This hydrophobic binding site plays a key role in the ligand recognition and selectivity. In addition, the aryl ring of Phe17 could have aromatic interaction with the active site Tyr and Trp so that the π–π interaction may be also an important factor that drives the binding. Our finding about the hot spot of Fab-peptide interface is consistent well with statistical analysis of the protein–protein interfaces that Arg, Tyr and Trp have the largest proportion [33,34]. In our complex, Glu and Asp are the hydrophilic complementary hot spot residues to Arg, while Phe is the hydrophobic one to Tyr and Trp.

### 2.2. Mutation

Through the MD simulations of the Fab-octadecapeptide system, it is found that the interaction between protein-ligand is mainly attributed to electrostatic interactions of charged residues, and hydrophobic residues also play a role in the binding. Interactions within the residues mentioned above (Glu2, Asp4, Pro16 and Phe17) exhibit a good selectivity and affinity. Mutation study in key residues that influence or even change the type of interaction was carried out to identify the interactions and residues that are important for Fab binding and some of which lead to an increase in the binding affinity.

To further elucidate the role of the electrostatic and hydrophobic interactions in the binding of the planar surfaces, a series of mutations were introduced into the system after analyzing the Fab-octadecapeptide interaction. First, a single point substitution mutation of key residues to glycine within the contact regions of the wild-type octadecapeptide was applied. The mutation would lead to the loss of side chain and confirm the roles of them in the interaction. Then, according to the properties of the side chain, we systematically mutated them to residues with different size, charge, hydrophobicity and/or polarity, and examined the effects on affinity and conformational specificity. Residues to be mutated and the definition of mutant are as follows: (1) Glu2 (the second residue of the octadecapeptide) to glycine (Glu2Gly) with no side chain, positively charged lysine (Glu2Lys), negatively charged aspartic acid (Glu2Asp) with a shorter side chain, serine (Glu2Ser) with a hydroxyl side chain; (2) Pro16 to glycine (Pro16Gly), the most hydrophobic residue tryptophan (Pro16Trp), hydrophobic tyrosine (Pro16Tyr) with hydroxyphenyl side chain; (3) Phe17 to glycine (Phe17Gly), tryptophan (Phe17Trp), tyrosine (Phe17Tyr), positively charged histidine (Phe17His) with imidazole side chain; (4) Asp4 to negatively charged glutamic acid (Asp4Glu) with a longer side chain, Pro16 and Phe17 by an exchange on substrate (Pro16Phe/Phe17Pro).

**Figure 5:**
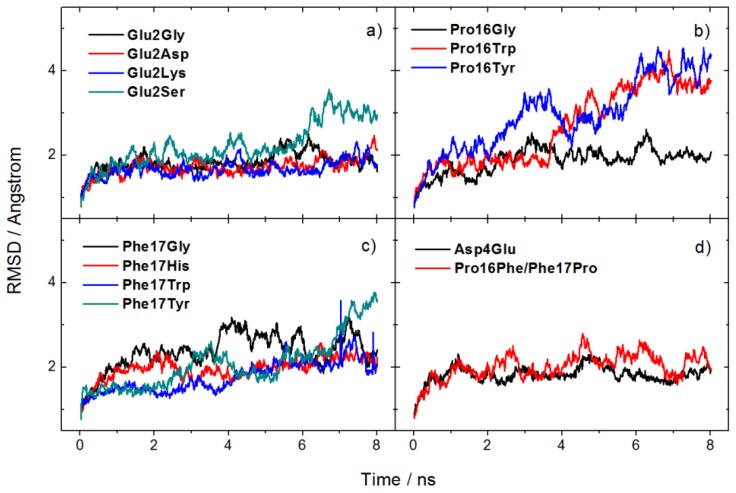
RMSD of mutated systems during (molecular dynamics) MD simulations.

Figure 5 shows the backbone RMSD of mutated peptides with the Fab complex system changing within runtime. In the simulations of the thirteen systems, glycine mutations usually caused little conformational changes with a small fluctuation less than 1 Å during the simulation time. However, an increase of RMSD value about 2 Å in which Glu2 is substituted with serine (Figure 5a) means significant conformational rearrangement caused by the fluctuation of the side chain, and then a backbone conformational rearrangement is needed. By comparison, Glu2Asp and Asp4Glu systems show both little conformation change and stable interaction because of the similar property and structure of side chains. The mutation of hydrophobic residues (Figure 5b,c) shows larger fluctuations especially for Phe16. It indicates that the binding mode should be well refined, because of the rearrangement of the binding site and ligands may affect the binding much, leading to some invalid designs. It reminds researchers of the importance of using the appropriate binding structure in structure-based ligand design with standard docking methods [24].

Thus, in order to understand how the mutation affects the interaction energy and how the hydrogen bonding changes, the interaction energies and hydrogen bond occupancies of peptides to Fab were calculated during the last 1 ns. The hydrogen bond term in Table 1 was determined by summing each probability of the hydrogen bonds (more than 10%), representing the average number of hydrogen bonds in each system. A significant decrease in interaction energy was observed for the octadecapeptide in which Glu2 was substituted by a glycine (Glu2Gly) or lysine (Glu2Lys) about 70 and 180 kcal·mol^−1^, respectively. This can be explained by the loss of strong electrostatic interactions between the side chain of Glu2 and arginine (Figure 4a), as well as the disappearing of hydrogen bonds from 3 to 1. Mutations of other residues (Pro16 and Phe17) to glycine did not reduce either the interaction energy or the hydrogen bonds much between octadecapeptide and Fab, which further demonstrates the importance of electrostatic interaction (Table 1). It is worth noting that when Pro16 or Phe17 was substituted with other hydrophobic residues, the ligand interaction to Fab markedly increased, especially in the Phe17Trp mutant with almost 100 kcal·mol^−1^. However, calculations on the interaction energy between single residue and Fab show that only a quarter of the increasing energy was contributed by the van der Waals interaction. It is therefore the effects of hydrophobic contacts on the electrostatic interaction of Glu2 and Asp4 that leads to the significant affinity augmentation. This indicates that hydrophobic residues play another important role in the binding for their determination of specificity. Another significant increase of interaction was found in the mutation Pro16Phe/Phe17Pro. It is interesting that the Pro16Tyr mutant provides greater affinity than the glycine and tryptophan mutations, which exhibit reduced affinity in the Phe17 substitution. It could be explained by the steric hindrance of side chains and the lip of the groove.

In general, it could be inferred that the hydrophobic tryptophan residues has a significant interaction with the whole hydrophobic groove (Figure 4c,d), leading to a significant enhancement of the binding. The spatial distribution of the four residues is in this order: Glu2, Asp4, Phe17 and Pro16. The high interaction hydrophobic tryptophan was then chosen as the terminal residue and it could be connected by a spacer group to the C-terminal of glutamic acid and spacer neighbored aspartic acid residue. In recent years, many small Fc-specific peptide ligands with high binding affinity have been successfully designed and synthesized such as tetrapeptide or hexapeptide for antibody purification [19,35]. Thus, a tetrapeptide ligand template Glu(E)-Asp(D)-spacer-Trp(W) was designed to generate potential affinity ligands. Residues showing the best enhancement in hydrophobic mutations especially at Phe17 were selected from the mutation study as the spacer group, including Trp, Gly and Pro. The ligand may retain the affinity interaction, the hydrogen bonds and the specificity of the binding.

### 2.3. Design and Evaluation of Tetrapeptide Ligands

**Figure 6:**
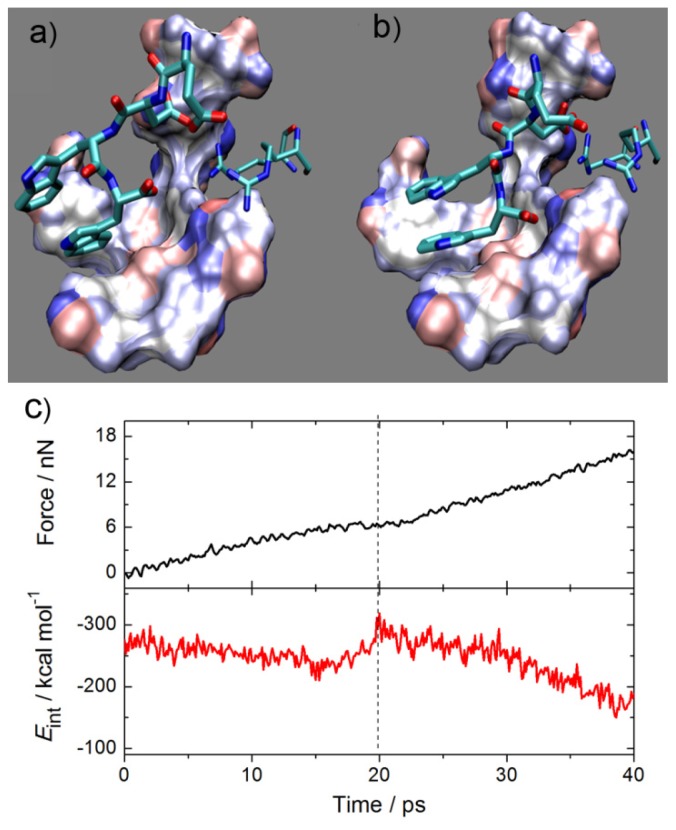
(**a**) and (**b**) are 0 and 20 ps snapshots of the EDWW-Fab system during the SMD simulation. The ligand EDWW is drawn as a stick model and colored by atom, the surface model colored by charge represents the Fab hydrophobic binding groove and the two thinner bond structures are Fab arginines; (**c**) The applied force and the interaction energy between ligand EDWW and Fab versus the simulation time.

Three tetrapeptide ligands, EDGW, EDPW and EDWW, were obtained by replacing the spacer group by potential connection residues, Gly (G), Pro (P) and Trp (W). The SMD simulation traces of tetrapeptide ligand entering the binding site on the Fab surface and the force and interaction curves versus time were extracted to find a tight complex. EDWW was taken as an example and shown in Figure 6. It can be observed that the ligand favorably drop into the cleft of the protein and it caused a small decrease of interaction energy during the first 15 ps. However, it is observed that a sharp increase of nearly 100 kcal·mol^−1^ in interaction energy occurred along with a flat force-time curve from around 15 ps, and there is a maximum absolute value of interaction energy of over 300 kcal·mol^−1^ at 20 ps, which could be accounted for by the formation of a tight Fab-ligand complex. In general, two atoms will feel Lennard–Jones repulsion at short distances. As the protein and ligand get closer from a position further than the equilibrium state, an attraction makes the slope of the external force versus time become progressively smaller and almost flat, so the interaction energy becomes larger. Once it exceeds the equilibrium position, a strong repulsion leads to a larger slope of the external force versus time and the ligand moves out of the binding site, as observed in the trajectories but not shown here, with a decrease in interaction energy. Hence, the tight complex structure of each system is confirmed by finding a scenario in which the slope of the external force versus time is around zero and the interaction energy reaches a salient point (around 20 ps in Figure 6c). Interaction energy (*E*_int_) and binding free energy (*ΔG*_bind_) of the three ligands to Fab from relaxation of the tight complex structure by MD simulations is shown in Table 2. It is observed that the tetrapeptide EDPW shows equivalent interaction energy to the wild type octadecapeptide. A more accurate prediction of binding affinity by free energy calculation suggests the same best binding ligand, EDPW. The two tryptophan (indole) residues of EDWW stack face-to-face first but move apart from each other because of the tension of the peptide backbone. For the EDGW system, the glycine with no side chain introduces more flexiblility and the interaction energy curve (not shown) has a sudden decrease. These phenomena may lead to the loss of affinity for these two ligands. Thus, EDPW has the best affinity and may be used as an affinity ligand for the purification of trastuzumab. It is also apparent that the proline ring can constrain and stabilize the conformation of the ligand well.

## 3. Experimental Section

In this study, all the simulations were performed using the NAMD program [36] with the Charmm27 force field [37] with isothermal–isobaric (NpT) ensemble. The parameters of Lennard-Jones potential for the cross interactions between non-bonded atoms (protein-water) were obtained from the venerable Lorentz–Berthelot combination rules [38]. A constant temperature of 298 K was controlled by a Langevin thermostat. The Langevin piston Nosé–Hoover method [39,40] was also employed to maintain a pressure at 101.3 kPa. The simulations were carried out with a time step of 2 fs, and a cut-off of non-bonded van der Waals interaction with a switching function starting at a distance of 12 Ǻ and reaching zero at 14 Ǻ. The Particle Mesh Ewald (PME) summation [41] was used to calculate long-range electrostatic interactions with a cutoff distance of 12 Å for the separation of the direct and reciprocal space. In all models, proteins were solvated in TIP3P (Transferable Intermolecular Potentials 3 Points) water molecules forming a box 10 Å bigger than the size of the protein, and some ions were added to electrically neutralize the system. Periodic boundary conditions were applied for all the simulations. Each system underwent 8 ns MD runs until the RMSD fluctuated around a constant value to reach the equilibrium state after a 5000-step energy minimization. The electrostatic potential was calculated by the algorithm developed by Zhang, *et al*. [42] based on a semiempirical quantum mechanical method Austin Model 1 (AM1) [43].

The time-dependent interaction energy, *E*_int_(*t*), for all the systems in MD and SMD simulations is defined similarly to our previous work [28]:

(1) $$E_{\text{int}}(t)=E_{\text{P}+\text{L}}(t)-E_{\text{P}}(t)-E_{\text{L}}(t)$$

in Equation (1)*E*_int_(*t*) stands for the interaction between the model protein and the ligand at time *t* during the MD or SMD simulation, and *E*_P+L_(*t*), *E*_P_(*t*) and *E*_L_(*t*) respectively refer to the total potential energy of the protein-ligand complex, the potential energy of protein and that of the ligand at time *t* during simulations. The electrostatic and van der Waals interaction energies were calculated in the same way. *E*_int_(*t*) is the quantitative indicator of the instantaneous interaction between the two molecules corresponding to each simulation moment, which is different from the interaction energy in general sense [44].

Three tetrapeptide ligands were designed rationally, then placed near their binding sites and equilibrated for 2 ns. Firstly, a better hydrophobic and electrostatic shape complementarity structure was established using PCV SMD pulling simulations processed once the equilibrium states were achieved. No atom was fixed in all MD simulations whereas backbone atoms of trastuzumab Fab were constrained in this SMD work. External force was applied uniformly on the backbone atoms of tetrapeptide ligands. The constant velocity *v* was fixed at 0.25 Å·ps^−1^ and the spring constant *k* was set to be 50 kcal·mol^−1^·Å^−2^ to obtain the best fit of SMD observation window and the force-time curves. The SMD simulation time was 40 ps and hence the center-of-mass moved about 1 nm. The direction of velocity and pulling force is from the backbone center-of-mass of ligand to that of trastuzumab. Then a tight-binding structure was obtained by analyzing the force-time and interaction energy-time plots. Finally, a relaxation of 8 ns MD simulation was carried out to reach the equilibrium state for free energy calculation. The protein-ligand binding free energies during the last 2 ns were calculated using the Prime/MM-GBSA module of Schrödinger suite [45] to get the averaged binding property. The binding free energy *ΔG*_bind_ was estimated using the equation [46]:

(2) $$\Delta G_{\text{bind}}=G_{\text{complex}}-(G_{\text{protein}}+G_{\text{ligand}})$$

where *G*_complex_ is the optimized free energy for the complex, *G*_protein_ and *G*_ligand_ are the optimized free energy for the free protein and free ligand, each energy term was calculated by a combination of molecular mechanics energy, implicit solvation energy and surface area energy. Residues in binding pockets of the protein were treated as flexible and the ligand partial charges were assigned by the initial Charmm charges.

## 4. Conclusions

In this work, the interaction mode between Fab of trastuzumab and a peptide fragment from HER2 was first investigated with MD simulations. Analysis of per-residue interaction energy, hydrogen bond, and conformation changes of these wild type Fab-peptide systems indicate that the interaction is concentrated in several key residues, consisting of Glu, Asp and Trp. In addition, the electrostatic interaction is the major contribution for affinity, while hydrogen bonds and hydrophobic interaction provides an orientation and specificity of the ligand. Thirteen mutation systems were set up based on this interaction mechanism analysis. These results show that interaction of the wild-type complexes could be increased significantly by mutation on the key residues, especially the system that Phe17 is substituted by a tryptophan. Consequently, three tetrapeptide ligands were designed rationally and evaluated based on the calculated energy from MD simulations and Prime MM-GBSA. Finally, the tetrapeptide EDPW shows an equivalent interaction energy to octadecapeptide when they are binding to transtuzumab. These results suggest that the investigation of binding mechanism is a targeted and effective approach for ligand design.

## Figures and Tables

**Table 1:** Interaction energies and hydrogen bond occupancies of peptides to Fab, where Glu2, Asp4, Pro16, Phe17 from the wild type peptide is substituted with residues in three-letter codes followed.

Mutation	Interaction energy (kcal/mol)	Hydrogen bond †
origin	−298.4 ± 12.7	3
Glu2Gly	−224.7 ± 18.0	1
Glu2Asp	−300.1 ± 21.0	2
Glu2Lys	−117.2 ± 17.8	0
Glu2Ser	−178.6 ± 20.4	1
Asp4Glu	−268.4 ± 16.6	2.5
Pro16Gly	−281.0 ± 23.2	3
Pro16Trp	−302.6 ± 16.1	2.5
Pro16Tyr	−254.7 ± 16.8	2.5
Phe17Gly	−310.7 ± 23.5	2.5
Phe17His	−294.6 ± 16.2	2.5
Phe17Trp	−396.6 ± 19.8	3
Phe17Tyr	−177.6 ± 16.9	3
Pro16Phe	−315.2 ± 18.0	3
Phe17Pro		

† Only considers probabilities of more than 10%.

**Table 2:** The energy contributions (kcal·mol^−1^) of tetrapeptide ligands to Fab.

Ligand	EDGW	EDPW	EDWW
*E*_int_	−262.0 ± 19.0	−295.1 ± 22.1	−190.1 ± 17.0
*ΔG*_bind_	−49.0 ± 4.8	−73.0 ± 6.2	−49.6 ± 5.3
